# Explainability of a Deep Learning Model for Mediastinal Lymph Node Station Classification in Endobronchial Ultrasound (EBUS)

**DOI:** 10.3390/bioengineering13020198

**Published:** 2026-02-10

**Authors:** Øyvind Ervik, Mia Rødde, Erlend Fagertun Hofstad, Thomas Langø, Håkon O. Leira, Tore Amundsen, Hanne Sorger

**Affiliations:** 1 Clinic of Medicine, Nord-Trøndelag Hospital Trust, Levanger Hospital, 7601 Levanger, Norway; hanne.sorger@ntnu.no; 2 Department of Circulation and Medical Imaging, Faculty of Medicine and Health Sciences, Norwegian University of Science and Technology, 7030 Trondheim, Norway; hakon.o.leira@ntnu.no (H.O.L.); tore.amundsen@ntnu.no (T.A.); 3 Department of Health Research, SINTEF Digital, Trondheim, 7034 Trondheim, Norway; mia.rodde@ntnu.no (M.R.); erlend.hofstad@sintef.no (E.F.H.); thomas.lango@sintef.no (T.L.); 4 HUNT Research Centre, Department of Public Health and Nursing, Faculty of Medicine and Health Sciences, Norwegian University of Science and Technology, NTNU, 7600 Levanger, Norway; 5 National Research Center for Minimally Invasive and Image-Guided Diagnostics and Therapy, St. Olavs Hospital, 7030 Trondheim, Norway; 6 Department of Thoracic Medicine, St. Olavs Hospital, Trondheim University Hospital, 7030 Trondheim, Norway

**Keywords:** endobronchial ultrasound (EBUS), artificial intelligence (AI)-augmented EBUS, deep learning (DL), artificial intelligence, explainable AI (XAI), lymph node classification, Grad-CAM

## Abstract

Accurate localization of thoracic lymph nodes during endobronchial ultrasound (EBUS) is crucial for lung cancer staging, treatment planning, and prognostication. Artificial intelligence (AI) has the potential to support this process. Deep learning (DL) models often lack transparency but can benefit from explainable AI (XAI) tools like Gradient-weighted Class Activation Mapping (Grad-CAM). However, no prior study has quantitatively assessed whether model attention in EBUS imaging corresponds to relevant anatomy. This study developed a convolutional neural network (CNN) to classify thoracic lymph node stations and evaluated the anatomical relevance of Grad-CAM activations using a structured annotation framework. Applied on 35,527 labeled EBUS images, the CNN achieved 63.1% accuracy, with the highest F1-score in stations 4L, 4R, and 10R. Three expert bronchoscopists independently annotated Grad-CAM maps from 3131 test images. Activations predominantly aligned with lymph nodes and/or blood vessels, yielding an accuracy of 65.9% and an F1-score of 58.4%, with moderate interobserver agreement. These findings indicate that DL can aid lymph node station classification and that XAI offers meaningful insight into model behavior. The proposed framework may enhance anatomical orientation and operator training during EBUS, although further optimization and multicenter validation are required.

## 1. Introduction

Lung cancer is the leading cause of cancer death worldwide, highlighting the urgent need for accurate staging to define the extent of disease and guide optimal therapy [1,2]. In the absence of distant metastases, endobronchial ultrasound (EBUS) is the method of choice to rule out or confirm cancer spread to regional lymph nodes, thus selecting patients eligible for curative therapies and a favorable prognosis [3,4]. EBUS is a minimally invasive technique in which an ultrasound bronchoscope is used to visualize mediastinal and hilar lymph nodes and to guide fine-needle sampling. Endoscopic ultrasound (EUS) via the esophagus complements EBUS by providing access to lymph nodes that are difficult to reach with the bronchoscope [5,6,7]. Mediastinal and hilar lymph nodes are categorized into stations, which are standardized anatomical levels numbered 1–14, based on the Mountain–Dresler classification and guidelines from the International Association for the Study of Lung Cancer (IASLC) [8,9,10,11]. Accurate identification of these stations is crucial for proper tumor–node–metastasis (TNM) classification and subsequent treatment decisions [1,2]. During lung cancer staging with EBUS, it is important to differentiate between hilar stations 10R and 10L and mediastinal stations 4L, 4R and 7, especially in patients who could be eligible for curative treatment strategies [10,11]. Distinguishing station 4R from 10R and 4R from 4L can be crucial to decide whether surgery is possible; however, the close anatomical proximity of these stations can make it technically challenging to distinguish them using EBUS. In addition, the interpretation of EBUS images is highly operator dependent, relying on the bronchoscopist’s training, experience, and institutional expertise [12,13,14].

Artificial intelligence (AI), particularly deep learning (DL), has the potential to enhance reproducibility and diagnostic performance in medical imaging [15,16]. AI-interpretation of EBUS images has mainly been used to segment anatomical structures and classify lymph nodes according to malignancy [17,18,19,20,21,22,23,24,25]. AI-based localization of lymph node stations during EBUS and EUS has hardly been explored [26,27]. Convolutional neural networks (CNNs) can automatically learn and recognize complex visual patterns in EBUS images, enabling real-time intraoperative classification of lymph node stations. This is a vital yet difficult task because of anatomical shifts, tissue displacement, and imaging artifacts [27,28,29,30]. However, a significant limitation remains because most AI systems act as “black boxes,” generating predictions without revealing the details of how the decisions were made [31]. This lack of transparency can significantly reduce clinical usefulness and trust in these methods. As a result, explainable AI (XAI) approaches have become a crucial strategy to enhance transparency, enabling clinicians to verify outputs and understand the reasoning behind automated classifications.

A central component of explainability is determining whether a model’s attention corresponds to clinically relevant anatomy [31]. XAI techniques, such as Gradient-weighted Class Activation Mapping (Grad-CAM), provide visual representations of the image regions that most strongly influence a model’s decision [32,33]. During conventional EBUS, bronchoscopists rely on grayscale ultrasound images to identify lymph nodes, blood vessels and surrounding tissue to orient the endoscope and guide needle sampling. Applying Grad-CAM to a model trained on EBUS images enables evaluation of whether the model’s attention is directed toward clinically relevant structures, mainly lymph nodes and blood vessels, or instead toward other structures or image artefacts [34]. Thus, demonstrating anatomically relevant model attention is essential for establishing reliable and clinically useful AI-augmented EBUS.

Recent studies have applied XAI in EBUS to visualize how DL models make diagnostic decisions [25,35]. Ishiwata et al. applied Grad-CAM to visualize model attention during lymph node malignancy classification, demonstrating that although activations frequently corresponded to lymph node structures, some predictions were influenced by regions outside the lymph nodes [25]. Lin et al. proposed TransEBUS, a hybrid DL model for distinguishing between malignant and benign mediastinal lymph nodes, and used Grad-CAM to visualize model attention [35]. They observed focused attention on lesions in correctly classified cases, whereas noise in the ultrasound images led to dispersed or inconsistent attention patterns, suggesting model confusion. Together, these findings indicate that model predictions may be influenced by structures outside lymph nodes or by image artefacts, raising potential clinical concerns by undermining model reliability and clinical trust in AI-assisted EBUS. However, these studies relied solely on visual, qualitative impressions of Grad-CAM maps. They have not quantified the degree to which the activations align with the underlying anatomy. Furthermore, prior use of AI in the field of EBUS has mainly focused on detection and malignancy classification tasks, while the classification of lymph node stations has received much less attention [26,27]. To date, to our knowledge, no published work has quantitatively examined AI-based lymph node station localization using XAI techniques.

In summary, this study offers three major contributions. First, we present a DL model for station-level classification of thoracic lymph nodes from prospectively acquired EBUS images, addressing a task that has received limited attention in previous EBUS-AI research. Second, we introduce a structured, quantitative framework for assessing explainability in EBUS by evaluating whether Grad-CAM activation maps align with clinically significant anatomical structures, such as lymph nodes and blood vessels. Third, we evaluate inter-observer agreement among expert bronchoscopists using standardized annotation guidelines, providing objective support of the anatomical plausibility and consistency of model attention. Together, these contributions have the potential to make AI-assisted EBUS a more interpretable, rigorous and clinically applicable method, marking an important step toward the integration of DL into bronchoscopy workflows.

## 2. Materials and Methods

### 2.1. Study Population

Patients scheduled for endobronchial ultrasound-guided transbronchial needle aspiration (EBUS-TBNA) due to enlarged thoracic lymph nodes were prospectively enrolled at two hospitals. Ethical approval was obtained from the Regional Committees for Medical and Health Sciences Research Ethics, Norway (identifier 240245, approval date 14 April 2021, and 588006, approval date 4 April 2023) and the Local Data Access Committee (identifier 2021/3210-19442/2021, approval date 21 June 2021, and 2023/1540-20710/2023, approval date 4 July 2023). The study was registered at ClinicalTrials.gov (identifier NCT05739331, approval date 22 February 2023).

### 2.2. Preoperative Preparations

All participants underwent standard clinical evaluation, including pulmonary function testing and contrast-enhanced chest and abdominal computed tomography (CT). When lung cancer was suspected, positron emission tomography with CT (PET-CT) was performed in patients who could be eligible for curative treatment.

### 2.3. Intraoperative Workflow

EBUS-TBNA was performed under conscious sedation following institutional guidelines. After airway inspection with a flexible bronchoscope, an Olympus BF-UC19OF scope (Olympus, Tokyo, Japan) connected to an EBUS processor (EU-ME2, Olympus) was used to examine mediastinal and hilar lymph nodes systematically [3,4]. Ultrasound images were recorded digitally, and lymph node stations (4L, 4R, 7, 10L, 10R, 11L, 11R) were labelled in real time according to the Mountain–Dresler classification and IASLC 8th and 9th editions [4,9,10,11,36,37]. The labelling of lymph node stations was performed by three expert bronchoscopists from two institutions, each having performed more than 500 EBUS procedures. To ensure consistency across the dataset, each image sequence was labeled once, in real-time, by a single expert bronchoscopist, namely the clinician performing the EBUS examination. Established anatomical landmarks were used for this purpose, adhering to the IASLC lymph node classification system. In this project, lymph node station 7 recordings were subdivided into 7R and 7L to differentiate imaging obtained from the right and left sides of the main carina at the first airway division. The study population, preoperative evaluation, and intraoperative EBUS workflow followed the protocol described in our previous publications [23,27].

### 2.4. Postoperative Image Processing

#### 2.4.1. CNN

**Figure 1:**
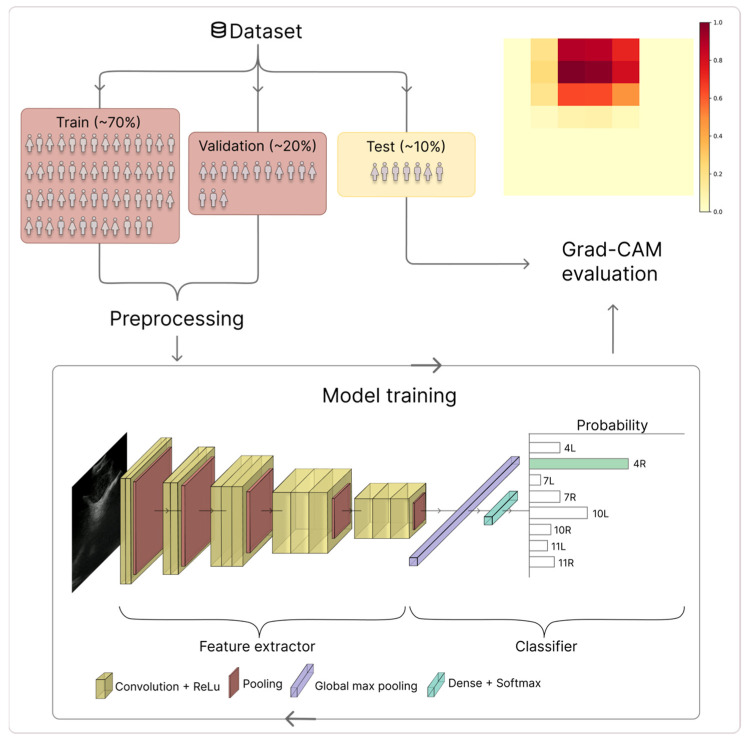
Overview of the study workflow, including patient-wise dataset splitting, preprocessing, model training, and Gradient-weighted Class Activation Mapping (Grad-CAM) evaluation. The figure illustrates the network architecture and classification pipeline, along with a representative Grad-CAM activation map used to assess model attention during the classification of lymph node stations. In the probability panel, the green box highlights the predicted class (i.e., the class with the highest probability). In the dataset boxes, different colors distinguish training, validation, and test subsets.

The labelled lymph node stations and corresponding EBUS images were used to train a DenseNet-121 CNN [38]. The model was implemented in TensorFlow/Keras and initialized with pretrained ImageNet weights [39]. The DenseNet-121 backbone was kept frozen during training, acting as a fixed feature extractor to reduce overfitting given the limited data. A lightweight classification head consisting of global average pooling, dropout (rate = 0.5) and a fully connected softmax layer with eight output units (corresponding to the number of lymph node stations) was added to enable robust prediction while limiting model complexity. This transfer-learning strategy allows the model to use general visual features learned from large-scale natural images while adapting to the target task. The EBUS images were resized to 224 × 224 pixels to fit the input dimensions of the network and preprocessed using the standard DenseNet normalization [38]. The dataset was split into training (70%), validation (20%), and test (10%) sets. Data augmentation was applied to the training set, including gamma transformation, blurring, rotation, contrast scaling, and Gaussian shadow augmentation (Figure 1). The model with the lowest validation loss across the training was saved. The hyperparameters to train the model are summarized in Table 1.

#### 2.4.2. Grad-CAM Generation

All EBUS image recordings in the test set were processed to generate Grad-CAM visualizations for expert assessment. The softmax (classification) layer of the model was removed, and the gradient information from the last convolutional layer (conv5_block16_concat) was used to assign activation weights to different image regions (Figure 1). This layer was selected because it preserves spatial resolution, while encoding high-level semantic features relevant to lymph node station classification [32].

**Figure 2:**
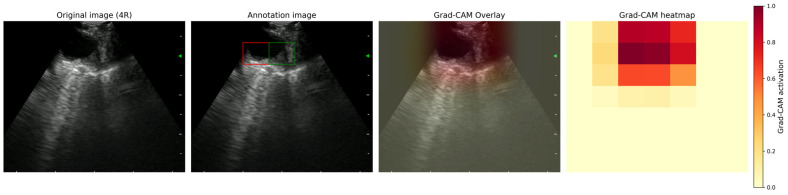
Example of Grad-CAM visualization for an EBUS image. (**Left**) Original ultrasound image of lymph node station 4R. (**Middle left**) The same EBUS image with an activation region (Grad-CAM ≥ 0.9). The square with the strongest activation is highlighted in red, and additional activated grid cells are shown in green. (**Middle right**) Grad-CAM overlay visualized on the original image, illustrating the spatial distribution of the model’s attention. (**Right**) Corresponding 7 × 7 Grad-CAM heatmap, where warmer colors indicate a stronger contribution to the model’s prediction.

Grad-CAM heatmaps were computed from the final convolutional layer and displayed as a 7 × 7 grid of semi-transparent colored squares overlaid on the corresponding ultrasound image (Figure 2). The grid-based representation was predefined and applied consistently throughout the study for structured expert annotation and quantitative evaluation. Additionally, continuous Grad-CAM heat maps were overlaid on the original EBUS images to enhance visual interpretation [41,42]. The activation region was defined as the combined area where Grad-CAM intensity was ≥0.9, with the grid cell exhibiting the highest activation value highlighted in red. The threshold of 0.9 was selected to ensure that only regions with high model confidence were included, thereby reducing noise and enabling expert evaluation to focus on the most prominent features. These visualizations served as the basis for assessing the model’s attention patterns. Since the Grad-CAM intensity was normalized to 1.0 for the cell with the highest activation, at least one cell was above the threshold in each image.

#### 2.4.3. Grad-CAM Activation Maps

To evaluate the model’s attention during classification of lymph node stations, we generated Grad-CAM activation maps for each EBUS image in the test set and analyzed the resulting patterns. The model produced a 7 × 7 activation grid. The maximum intensity square, defined as the grid cell with the highest activation value, was identified. This square indicated the area that contributed the most to the lymph node station classification of that image. The x and y coordinates of the max-intensity square were extracted for every image and grouped by lymph node station. For each station, a two-dimensional frequency map was created by counting how often the max-intensity square appeared in each of the 49 grid positions. These distributions were visualized as Grad-CAM activation heatmaps to illustrate characteristic localization patterns within individual stations and overall trends across the dataset.

#### 2.4.4. Expert Annotation of Grad-CAM Heatmaps

**Figure 3:**
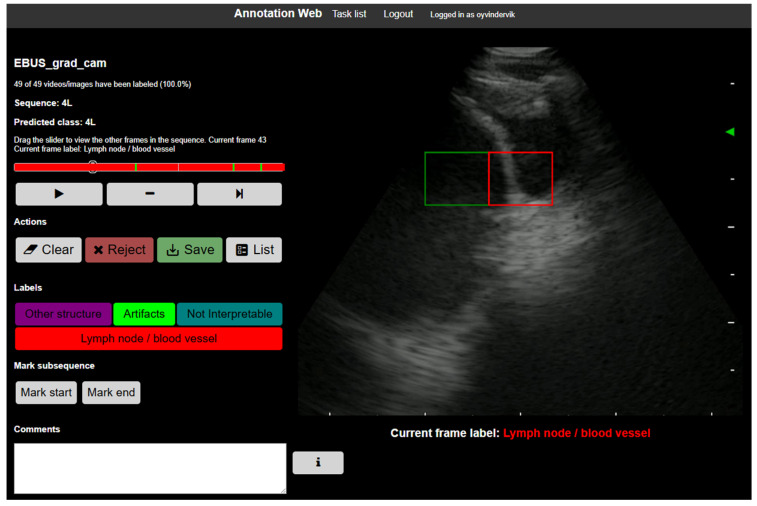
Screenshot of the software interface used for expert labeling of Grad-CAM activation regions [43]. The red-highlighted grid cell represents the maximum activation point, while green squares indicate additional cells within the activation region. In this example, the highlighted activation area overlaps with clinically relevant landmarks in lymph node station 4L, specifically the pulmonary artery and a lymph node.

Three expert bronchoscopists independently annotated each Grad-CAM visualization according to a standardized guideline developed explicitly for this study (Supplementary Materials S1). Annotations were performed and documented using the open-source platform AnnotationWeb (Figure 3) [43]. All evaluations were performed on individual images, assessed independently without reference to preceding or subsequent frames. For every EBUS image, annotators identified the activation region and assigned one of four predefined labels: lymph node/blood vessel, other structure, artifact, or not interpretable.

The anatomical relevance of the model’s activation was assessed manually by determining whether the activation region, defined as the combined set of all Grad-CAM grid squares with an activation value of ≥0.9, overlapped with clinically meaningful anatomical structures. All squares meeting this threshold were regarded collectively as a single activation region, regardless of their number or location. The four annotation categories were selected to represent the image features most relevant to EBUS interpretation. During EBUS, identifiable vessels and lymph nodes are the main anatomical landmarks used to distinguish lymph node stations and guide appropriate sampling; activations in these areas were therefore considered clinically relevant. Activations corresponding to recognizable ultrasound artifacts were labeled as “artifact,” since such patterns may cause misleading image interpretation. In clinical practice, however, artifacts such as acoustic shadowing or reverberations may occasionally be of value to confirm the position of the EBUS probe. Activations overlying the airway wall, cartilage, lung parenchyma, or background tissue were categorized as “other structure”. A “not interpretable” label was assigned when no coherent anatomical interpretation could be made, typically due to poor-quality or ambiguous images. All assessments were performed with reference to the whole ultrasound sector to ensure that activation patterns were interpreted in a proper anatomical context.

#### 2.4.5. Quality Control and Consensus

The annotation guidelines were created and refined iteratively in cross-disciplinary meetings involving the three expert bronchoscopists and one AI expert. All participants agreed upon the final version of the annotation guideline. Before starting individual annotations, all three annotators participated in two consensus sessions in which 391 example images, drawn from a single, representative patient and spanning seven lymph node stations, were jointly reviewed to ensure consistent interpretation and uniform application of the annotation criteria. During the evaluation of inter-observer agreements, designated annotation tasks were created in AnnotationWeb [43], and the annotators were blinded to one another’s assessments.

Inter-observer agreement was evaluated using kappa statistics, calculated exclusively from the independent annotations of each expert bronchoscopist prior to any consensus adjudication. All consensus labels were generated after the kappa analysis and were not included in the calculation of inter-observer agreement. Kappa values were computed based on predefined Grad-CAM attention categories, which included lymph node/blood vessel, other structure, artifact, and not interpretable.

If two annotators had assigned the same label, that label was accepted as the final classification. In images where all three annotators disagreed, the discrepancies were discussed in a consensus meeting where a collective agreement was determined.

## 3. Results

**Figure 4:**
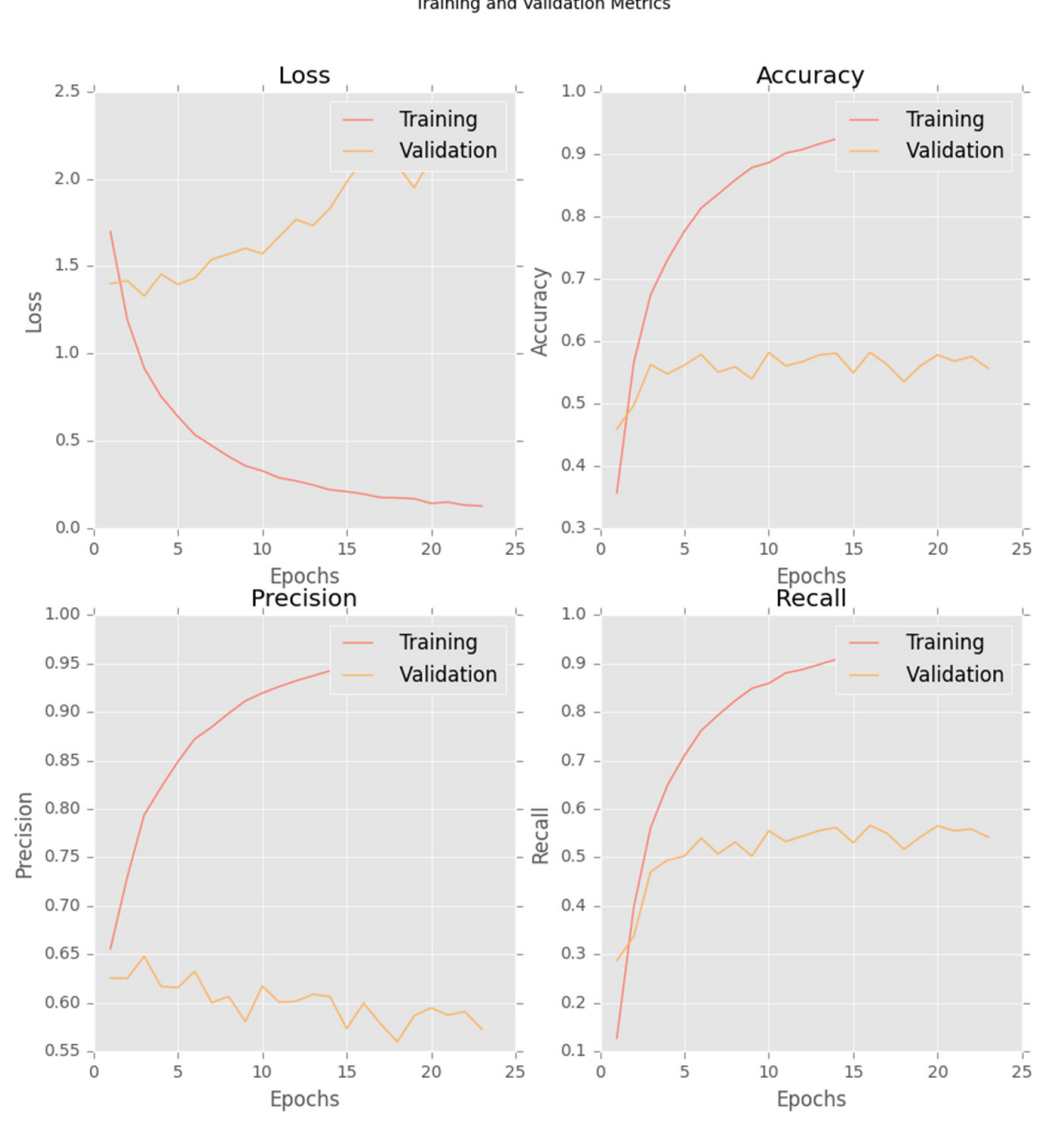
Training and validation learning curves for the DenseNet-121 model, showing loss, accuracy, precision, and recall across epochs.

Model training was monitored using loss, accuracy, precision, and recall on both the training and validation sets. The final model was selected based on the lowest validation loss, which occurred at epoch 3. Early stopping with a patience of 20 epochs was applied. The corresponding learning curves are shown in Figure 4.

### 3.1. Dataset and CNN Classification Performance

**Figure 5:**
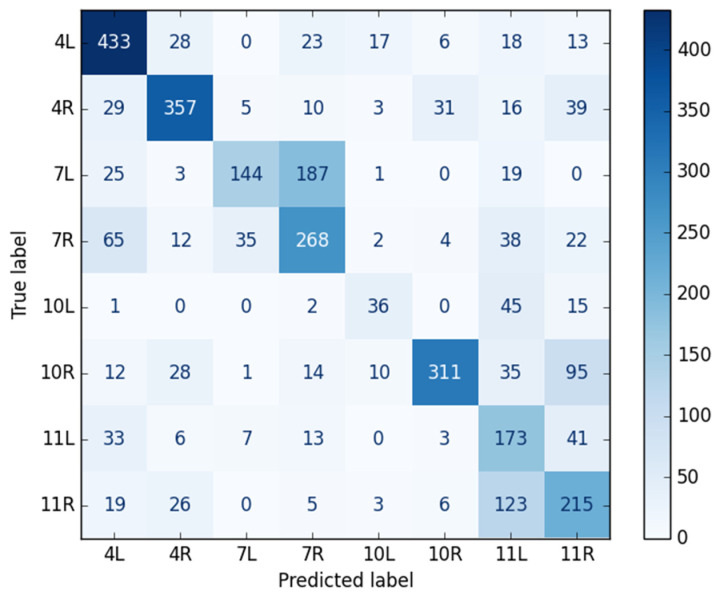
Confusion matrix of lymph node station classification. Rows represent true labels; columns represent predicted labels. Color intensity indicates the number of samples per class. The matrix illustrates class-wise performance and misclassification patterns across lymph node stations.

A total of 35,527 EBUS images were collected from 75 patients. The test dataset included 3131 images from seven patients, all of which were incorporated into the analysis. The CNN achieved an overall accuracy of 63.1%, with a precision of 62.8%, a sensitivity of 59.0%, and an F1-score of 59.1% across all lymph node stations. Table 2 displays the model’s performance values for each lymph node station. Figure 5 presents the confusion matrix for lymph node station classification, showing true versus predicted labels across all classes.

Table 3 presents both macro-averaged and weighted-averaged performance metrics. The macro-averaged scores assign equal weight to each lymph node station, highlighting consistent performance across stations. In contrast, the weighted-averaged scores account for each station’s relative frequency in the dataset. Reporting both metrics provides a more complete and representative assessment of the model’s overall performance.

### 3.2. Grad-CAM Activation Patterns

**Figure 6:**
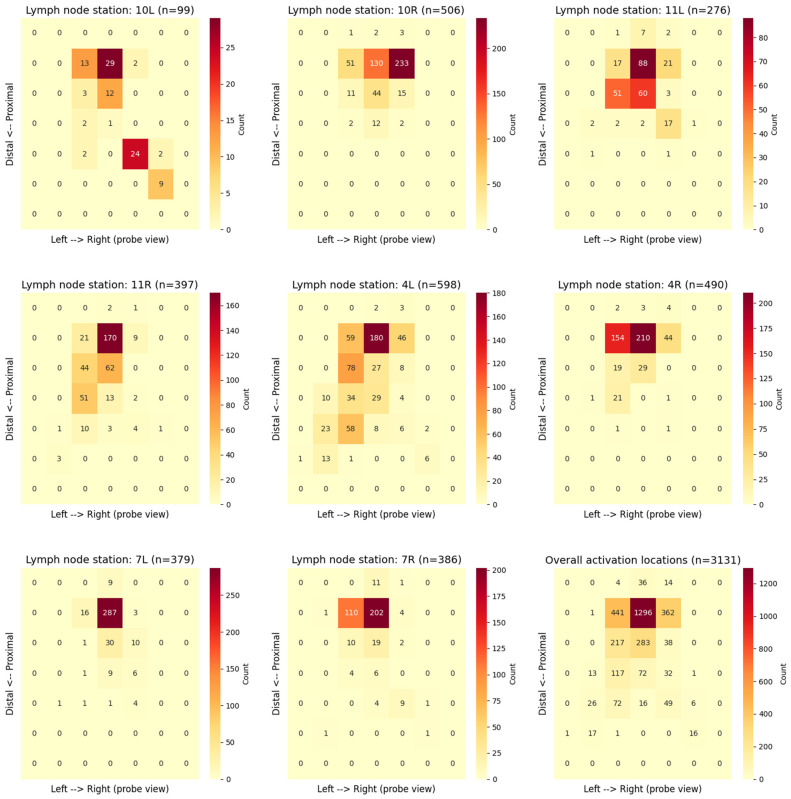
Heatmaps illustrating the distribution of maximum-intensity locations from the 7 × 7 Grad-CAM activation grid for each lymph node station, with the overall distribution across all stations displayed in the bottom right.

For each image, the highest-activation square from a 7 × 7 Grad-CAM grid was extracted to characterize the model’s attention distribution across lymph node stations (Figure 6). The resulting heatmaps illustrate the spatial distribution of maximum activations within the ultrasound sector. The horizontal axis corresponds to the probe’s left–right orientation, while the vertical axis reflects the distal–proximal direction relative to the ultrasound transducer. Color intensity indicates the frequency of maximum activations in each grid cell, with darker red denoting higher occurrence. The station-specific heatmaps showed distinct patterns of localization, reflecting anatomical differences and probe orientation variations between stations. The bottom-right panel of Figure 6 displays the overall activation locations heatmap, combined across all stations (3131 images), which clearly indicates that the model tends to focus on the central–proximal region of the probe view.

### 3.3. Inter-Observer Annotation Agreement

#### 3.3.1. Annotation Workflow

**Figure 7:**
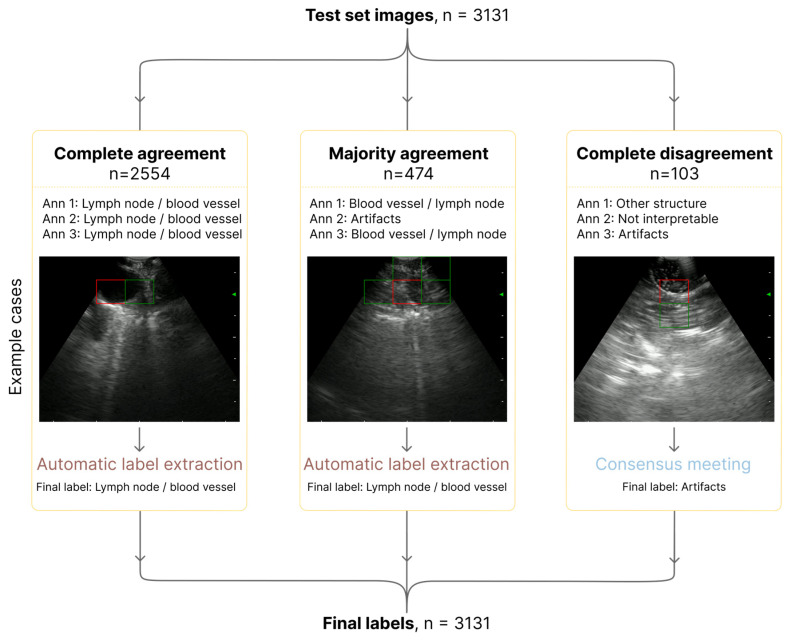
Annotation workflow and consensus process. The left example illustrates complete agreement among all three annotators, shown by an image in which the blood vessel is clearly identifiable. The middle example demonstrates majority agreement, for an image with small lymph nodes, motion artifacts, and reverberations; in this case, the label selected by two of the three annotators was automatically accepted as the final label. The right example shows complete disagreement, where each annotator assigned a different label (other structure, interpretable, or artifact). After a dedicated consensus meeting, the example image was labeled as artifact. Red squares indicate the grid cell with the highest Grad-CAM activation, while green squares represent additional neighboring cells included in the activation region. Text in dark red denotes automatic label extraction, and blue text indicates the consensus meeting used to determine the final label cases of complete disagreement.

All three experts from two institutions independently annotated 3131 EBUS images. The distribution of agreement levels is shown in Figure 7. Complete agreement among the annotators was achieved for most images (2554, 81.6%), whereas majority agreement was achieved for 474 images (15.1%). Only 103 images (3.3%) resulted in complete disagreement and were later reviewed in a consensus meeting, where a final label was assigned.

#### 3.3.2. Agreement Results

Inter-observer reliability was moderate, with an overall agreement of 81.6% and a Fleiss’ kappa of 0.529. Pairwise agreement between annotators was consistent, with Cohen’s kappa values ranging from 0.473 to 0.567 (Table 4).

### 3.4. Anatomical Relevance of the Model’s Attention

A total of 3131 Grad-CAM maps were annotated. The anatomical relevance of the model’s attention was assessed across expert-annotated categories. The overall CNN performance scores for the images of each category are presented in Table 5.

## 4. Discussion

This study demonstrated that DL-based classification of lymph node stations could be combined with XAI techniques to improve model interpretability in the context of EBUS imaging. While previous research has primarily focused on DL in malignancy prediction or segmentation tasks, this work presented a structured, quantitative assessment of Grad-CAM activations for lymph node station classification [17,18,19,20,21,22,23,24,25,27,35].

We addressed a critical methodological gap by quantifying inter-observer agreement and the model’s interpretability through assessment of Grad-CAM activation patterns. Our findings showed that a substantial proportion of Grad-CAM activations aligned with clinically relevant structures. These results underscore the importance of transparency in AI-augmented ultrasound bronchoscopy, where explainability tools could enhance clinical trust and support practical applications in training, quality assurance, and methodological refinement of future systems.

The proposed DL model achieved an overall accuracy of 63.1%, with moderate precision and sensitivity across stations. The difference between the macro-averaged F1-score (59%) and the weighted F1-score (63%) highlighted the effect of class imbalance on model performance (Table 3). The weighted metrics reflected the performance of well-represented stations, such as 4L, 4R, and 10R, where the model performed well. In contrast, the macro average showed lower performance at underrepresented stations, such as 10L and 11L. This distinction underscored the importance of a balanced dataset for effective station-level classification. Performance varied significantly by anatomical location, with lymph node stations 4L, 4R, and 10R attaining the highest F1-scores (Table 2). The superior performance of these stations was probably attributed to their more distinctive sonographic appearance and greater representation within the dataset. Conversely, stations such as 10L, 7, 11L, and 11R proved more challenging for the model. The suboptimal performance at 10L was consistent with the well-recognized technical difficulty of locating and clearly defining this station during EBUS, as well as its underrepresentation in the dataset [44,45]. Furthermore, the model struggled to distinguish between left and right at stations 7 and 11, which was understandable given the indistinct sonographic landmarks and the bilateral symmetry of these regions [4]. However, for the bronchoscopist working distal to the carina at the first airway division, recognizing right from left should be straightforward because visual control is available simultaneously through video bronchoscopy.

We have not been able to identify published studies that have specifically evaluated station-level classification using XAI. Thus, a direct comparison to prior research on EBUS AI is challenging. The only directly comparable work was our group’s previous publication, which utilized a CNN (MobileNetV3) combined with a long short-term memory (LSTM) network and achieved an accuracy of 59.5% through the incorporation of temporal information [27]. In contrast, the current image-based CNN model (DenseNet-121) achieved slightly better performance (63.1% accuracy), likely due to a larger and potentially more diverse dataset and the CNN architecture. Although research in related clinical fields, such as the EUS-MPS system, has demonstrated the feasibility of station localization, the method benefited from a more consistent imaging environment in the gastrointestinal tract and pre-filtered, higher-quality images [26]. In contrast, EBUS imaging was often affected by noise, respiratory motion, and acoustic artifacts, underscoring the need for AI solutions tailored to the specific challenges of airway ultrasound.

The current classification performance is moderate and must be further improved before clinical implementation as a fully autonomous AI tool. Nevertheless, at this stage, the proposed method is primarily intended as an AI-augmented support tool to assist with anatomical orientation and clinical decision-making during EBUS. Importantly, the model demonstrates stronger performance for several well-represented and clinically important lymph node stations, including 4L, 4R, 7, and 10R, as shown in the class-wise performance metrics (Table 2) and the confusion matrix (Figure 5). In lung cancer, accurate differentiation among these closely adjacent stations is particularly important for mediastinal staging and subsequent treatment planning.

A key contribution from this study was the quantitative assessment of the anatomical relevance of the model’s attention, advancing beyond the predominantly qualitative XAI assessments reported in previous EBUS studies. For images in which Grad-CAM showed the model’s activations focused on lymph nodes and/or blood vessels, the model achieved stronger localization performance, with an accuracy of 65.9% and an F1-score of 58.4%. These findings indicated that the model tended to focus on structures relevant to clinical decision-making, such as distinct vessels and lymph nodes, which clinicians rely on to define lymph node stations and guide appropriate sampling. Conversely, performance declined considerably in categories where the activation region focused on artifacts or non-interpretable images, resulting in an F1-score of less than 41%. The particularly low accuracy of 21.1% for the “other structure” category, including activation in the airway wall or lung parenchyma, suggested that the model exhibited near-random localization when the activation occurred in anatomical structures outside the lymph node or adjacent vessels. The moderate interobserver agreement across annotators, reflected by a Fleiss’ kappa of 0.529, underscored both the inherent subjectivity of ultrasound interpretation and the significant variability in EBUS image appearance across patients, anatomical regions, and operators. However, the high rate of complete agreement among all three annotators (81.6% of images) underscored the reliability of the standardized annotation guidelines and the overall interpretability of the evaluations.

Methodologically, we chose to distinguish four anatomical activation categories rather than merging “other structure”, “artifact”, and “not interpretable” into fewer labels. Although collapsing these categories into a binary scheme (lymph node/blood vessel vs. other) might have reduced annotation noise and improved interobserver agreement and performance metrics, this simplification would have obscured relevant patterns of incorrect model attention. The more granular categorization provided deeper insight into how and where the model failed, which is essential to understand its limitations and guide future improvements. We acknowledge that certain categories were underrepresented, warranting cautious interpretation of the results for these groups. Accordingly, the analyses are presented as descriptive rather than definitive, and confirmation of these observations will require larger, more balanced datasets.

The localization analysis of the 7 × 7 Grad-CAM grids provided a quantitative characterization of the model’s attention patterns during lymph node station classification. The overall activation localization heatmap (bottom-right panel of Figure 6), derived from 3131 images, indicated that the model mostly focused on the central-proximal region of the ultrasound sector. This tendency aligned with the inherent characteristics of the EBUS field of view, where image resolution is highest near the probe, and lymph nodes are typically located in close proximity to the transducer. The station-specific heatmaps (Figure 6) revealed localization tendencies that correspond well with known anatomical landmarks. Notably, in stations 4L, 4R, and 10R, the model’s attention focused on lymph node regions and major vascular structures, such as the pulmonary artery, ascending aorta, azygos vein, and superior vena cava. This concentration suggested that the model successfully learned meaningful spatial patterns relevant to anatomical orientation, rather than relying on non-diagnostic image cues. Conversely, more dispersed activation patterns were observed in stations 11L, 11R, and 10L. This dispersal reflected the greater anatomical variability in these locations and the specific issue of underrepresentation in the dataset for station 10L. Overall, these findings demonstrated anatomically plausible localization behavior, reinforcing the potential of Grad-CAM as a validation tool to confirm that the model was concentrating on the intended target structures rather than on artifacts.

This study had several notable strengths. First, the EBUS videos were collected prospectively in a real-world clinical setting, ensuring the dataset reflects the variability, artifacts, and challenges inherent in routine practice rather than being based on highly selected cases. Second, we established a robust reference standard by having three experienced bronchoscopists from two institutions independently annotate all the Grad-CAM images. This process used a carefully developed guideline, maintained blinding, and implemented a structured consensus workflow for cases of complete disagreement, thereby enhancing the reliability of the reference standard. Third, we conducted a detailed quantitative analysis of model activation patterns, which represents a significant methodological advance beyond the predominantly qualitative XAI assessments reported in previous EBUS studies.

However, some limitations should be acknowledged. First, even though two institutions participated in the study, the entire dataset was collected by three bronchoscopists, using an ultrasound processor and EBUS-bronchoscope from the same manufacturer. This approach may restrict the generalizability of the results to other operators, equipment, and clinical settings. Furthermore, certain lymph node stations were underrepresented in the dataset, which may have influenced the model’s overall performance; however, this imbalance is unlikely to represent a systematic bias.

From a methodological perspective, the choice of Grad-CAM implementation warrants consideration. We chose the final convolutional layer for Grad-CAM interpretation because it provides an optimal trade-off between semantic relevance and spatial localization. Earlier layers primarily capture low-level textures, whereas later pooling and fully connected layers lack spatial detail. This balance makes the final convolutional layer particularly suitable for visualizing model attention in EBUS images [32].

Methodological limitations include the constraint of the 7 × 7 Grad-CAM resolution used in the analysis, which limits detailed localization. Additionally, our evaluation was based on individual images rather than video sequences, meaning it lacked the temporal context that clinicians routinely consider during clinical decision-making. The decision to set a high activation threshold (≥0.9) was intended to ensure clearly interpretable attention regions suitable for structured expert annotation; however, this conservative choice may have excluded moderate-level activations that could contain additional, potentially informative features. Finally, despite two consensus sessions, some subjectivity remains in the annotations, especially in medium to low quality images, which is unavoidable in real-world ultrasound assessment.

To overcome the limitations acknowledged in this study, future research should explore several complementary directions. From a technical perspective, expanding the dataset’s size and diversity through multi-center collaborations will be vital to improving model robustness and generalizability. In addition, improving class balance and incorporating temporal information into the model architecture may better capture the contextual cues in video sequences that clinicians routinely rely on during decision-making. Implementing image-quality filtering could help remove images unlikely to yield reliable predictions. Additionally, methodological advances such as higher-resolution or multi-scale attention mechanisms, including applying Grad-CAM to earlier convolutional layers, Grad-GAM++ [46], which extends Grad-CAM to provide improved localization, or layer aggregation, could be pursued to improve model interpretability beyond the 7 × 7 Grad-CAM resolution used here. From a clinical standpoint, integrating real-time explainability into the EBUS workflow alongside station classification and segmentation models may assist with bronchoscope navigation, guide needle sampling, and support bronchoscopy training. Finally, ensuring that these AI tools remain simple, fast, and accessible will be crucial to their adoption in everyday practice. The annotation guidelines and evaluation framework provided in this work can serve as a foundation for standardized, reproducible research and will have transfer value to explainable AI applications in other ultrasound-based procedures.

XAI is essential in EBUS because accurate identification of and sampling from lymph node stations directly influences cancer staging and treatment decisions. Although Grad-CAM does not directly improve classification accuracy and is not intended to be part of the clinical diagnostic decision-making process, it plays a critical role in supporting the validation and safe development of AI-assisted EBUS systems. By visualizing the image regions that drive the model’s predictions, Grad-CAM enables assessment of anatomical plausibility and identification of unreliable or ambiguous outputs, thereby supporting quality assurance and iterative model refinement.

As AI-based tools are increasingly explored for integration into EBUS workflows, such transparency will be vital to build trust, ensure safety, and support future clinical implementation.

## 5. Conclusions

This study introduced a structured and quantitative framework for the explainability of AI-augmented EBUS. We demonstrated that DL could classify thoracic lymph node stations, and that Grad-CAM provided meaningful, anatomically relevant explanations for the model’s decisions. Clinically, the explainability framework supported anatomical orientation, enhanced transparency in decision-making, and may have contributed to training and quality assurance in EBUS diagnostics, particularly in lung cancer staging. In practice, the method represented a simple, fast, and low-cost solution that could be integrated into existing EBUS workflows with minimal burden. Although further optimization and multicenter validation are necessary, this work marked a promising step towards trustworthy, clinically relevant AI-augmented support during EBUS.

## Figures and Tables

**Table 1:** The hyperparameter values used to train the deep learning models.

Hyperparameter	Value
Epochs	100
Batch size	32
Optimizer	AdamW [40]
Loss function	Categorical cross-entropy
Learning rate	1 × 10^−4^
Patience	20

**Table 2:** Precision (positive predictive value), sensitivity (recall) and F1 score metrics for the classification model across all lymph node stations.

Lymph Node Station	Precision (%)	Sensitivity (%)	F1-Score (%)	Number of Images
4L	77.6	81.6	79.5	598
4R	75.6	72.7	74.1	490
7L	76.6	38.8	51.5	379
7R	52.1	69.9	59.7	386
10L	50.7	36.4	42.4	99
10R	85.8	59.9	70.5	506
11L	36.2	60.9	45.4	276
11R	47.8	52.1	49.9	397
Total				3131

**Table 3:** Macro-averaged and weighted-averaged precision, recall, and F1-score for the classification model across all lymph node stations.

Metric	Precision (%)	Sensitivity (%)	F1-Score (%)	Number of Images
Macro average	62.8	59.0	59.1	3131
Weighted average	67.1	63.1	63.5	3131

**Table 4:** Inter-observer agreement metrics for the three expert annotators.

Agreement Metric	Annotator Pair	Value	Interpretation
Percent agreement	All annotators	81.6%	–
Fleiss’ kappa	All annotators	0.529	Moderate agreement
Cohen’s kappa	Annotator 1–Annotator 2	0.598	Moderate agreement
Cohen’s kappa	Annotator 1–Annotator 3	0.531	Moderate agreement
Cohen’s kappa	Annotator 2–Annotator 3	0.474	Moderate agreement

**Table 5:** Overview of CNN performance metrics stratified by expert-annotated attention categories, including accuracy, precision, sensitivity, and F1 score.

Label	Accuracy (%)	Precision (%)	Sensitivity (%)	F1-Score (%)	Number of Images
Lymph node/blood vessel	65.9	61.7	59.5	58.4	2715
Artifacts	45.9	47.6	40.6	40.4	196
Not interpretable	45.3	32.8	29.9	29.6	201
Other structure	21.1	45.8	48.3	34.2	19
Total					3131

## Data Availability

The datasets generated and/or analyzed in the current study are not publicly available since they are part of an ongoing study. Data might be available upon reasonable request, on a mutual collaborative basis. Please contact the first author/Øyvind Ervik.

## Supplementary Materials

The following supporting information can be downloaded at: https://www.mdpi.com/article/10.3390/bioengineering13020198/s1, Supplementary Material S1: Grad-CAM annotation guideline for the clinician (Supplementary material).

## Abbreviations

AI	Artificial intelligence
CNN	Convolutional neural network
DL	Deep learning
EBUS	Endobronchial ultrasound
EBUS-TBNA	Endobronchial ultrasound-guided transbronchial needle aspiration
EUS	Endoscopic ultrasound
Grad-CAM	Gradient-weighted Class Activation Mapping
IASLC	International Association for the Study of Lung Cancer
LSTM	Long short-term memory
CT	Computed tomography
PET-CT	Positron emission tomography–computed tomography
TNM	Tumor–node–metastasis
XAI	Explainable artificial intelligence
