# Supplementary material for: Explainability of a Deep Learning Model for Mediastinal Lymph Node Station Classification in Endobronchial Ultrasound (EBUS)
Source: Bioengineering (Basel). 2026 Feb 10;13(2):198. doi: 10.3390/bioengineering13020198 (PMC12938215; doi:10.3390/bioengineering13020198)
Supplement: Supplementary file 1 [file bioengineering-13-00198-s001.zip › bioengineering-4101343-supplementary.pdf]

Grad-CAM annotation guidelines for the clinician (Supplementary material)

1. Scope

This Supplementary Material provides comprehensive guidelines for annotating and evaluating Gradient-weighted Class Activation Mapping (Grad-CAM) heatmaps in artificial intelligence (AI) for Endobronchial Ultrasound (EBUS).

The goal of Grad-CAM annotation is to quantitatively evaluate how well the model's attention areas align with clinically relevant anatomical structures, such as lymph nodes and blood vessels, in EBUS images. Standardizing this process supports consistent, reproducible evaluation of model explainability across annotators.

To support this goal, the following section explains how Grad-CAM visualizations are generated, how annotations are performed, and how annotation decisions are made.

2. Description of Grad-CAM visualizations

A Grad-CAM map visualizes the regions of an image that contribute most strongly to the model's decision. In this project, Grad-CAM heatmaps are generated from EBUS images and displayed as **semi-transparent square overlays** on the original ultrasound images (Figure S1).

Annotations are performed to determine whether the **activation region** (all grid squares with Grad-CAM intensity ≥ 0.9) corresponded to relevant anatomical structures.

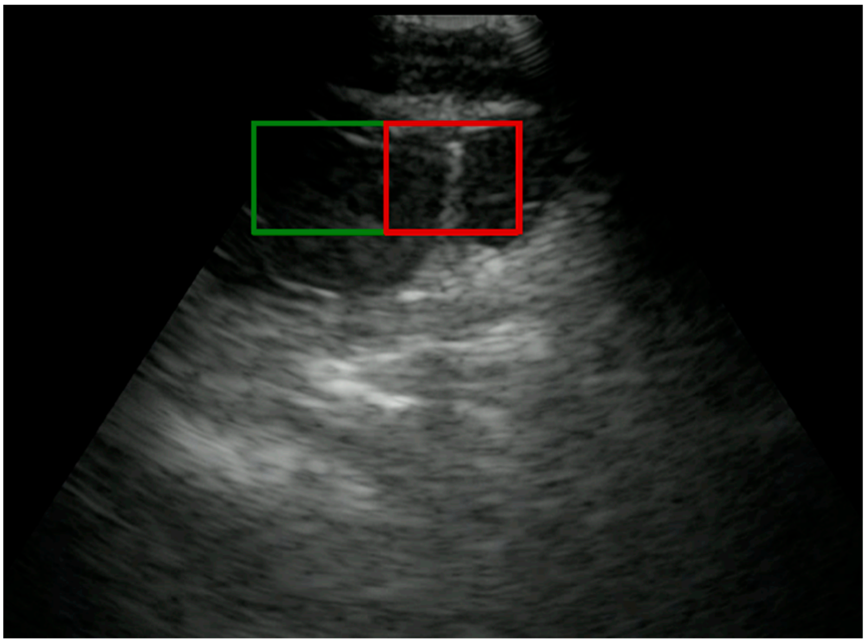

Figure S1: Example of Grad-CAM visualization used for expert annotation.
The original EBUS image displays semi-transparent square overlays that denote the Grad-CAM activation region (activation intensity ≥ 0.9). The grid cell with the highest activation value, highlighted in red, marks the max-activation region used for annotation. Green squares indicate additional grid cells encompassed within the activation region.

3. Annotation steps

For each Grad-CAM visualization, annotators should adhere to a standardized, step-by-step protocol to ensure consistent and reliable interpretation.

Annotators should:

3.1. Identify the activation region.

3.2. Evaluate the activation region in the context of the entire ultrasound sector.

3.3. Determine whether this activation region overlaps a clinically relevant structure (lymph node and/or blood vessel).

3.4. Assign one primary label per image based on the activation region:

3.4.1. Lymph node / Blood vessel

- If the activation covers a clinically relevant structure, such as lymph nodes and/or blood vessels.

3.4.2. Artifact

- If the activation aligns with technical artifacts such as acoustic shadow, reverberation, motion, or noise.

3.4.3. Other structure

- If the activation lies outside any lymph node/ blood vessel (e.g., cartilage, airway wall, lung, or background).

3.4.4. Not interpretable

- If the image quality is globally degraded and no anatomical structures can be confidently identified.

Representative examples for each annotation category are provided in Section 7 (*Examples of Annotations).*

To further ensure consistency, a set of explicit annotation rules should be applied, as described below.

4. Annotation rules:

4.1. Evaluate each image independently

- Annotations must be based solely on the visual content of the single image, without considering the previous or subsequent images.

4.2. Clinically relevant structures take priority

- Label the activation region as *Lymph node / Blood vessel* if it lies within or touches even a small part of a lymph node or vascular structure.

4.3. Overlapping structures

- If the activation region overlaps multiple structures, select *Lymph node / Blood vessel* whenever any overlap with these structures exists, even if minimal.

4.4. In the absence of a lymph node or blood vessel, artifacts outweigh other structures

- If the activation region overlaps other structures, but any part of the activation corresponds to an artifact, label the image as *Artifact*.

4.5. Ambiguous cases:

- If uncertainty remains after applying the above criteria, the label corresponding to the visually dominant activation region shall be assigned.
- If no dominant anatomical or artifact can be identified, the image shall be labeled *Not interpretable.*

When uncertain whether an image should be labelled Not interpretable or Artifact, the following rules should be applied:

5. Artifact vs. not interpretable

Table S1: Criteria for Classifying Artifact and Not Interpretable images.

Criteria	Artifact	Not interpretable
**Description**	Localized image degradation where some anatomical structures remain visible	Global or severe degradation; no identifiable anatomical structures
**Extent of issue**	Affects a limited region of the ultrasound sector	Affects most of **the ultrasound sector**, preventing meaningful interpretation
**Interpretability**	Partly usable for interpretation and orientation	Not usable; anatomical structures and orientation cannot be determined
**Label**	Artifact	Not interpretable
**Example image**	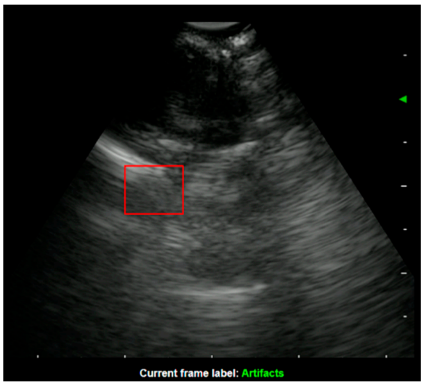	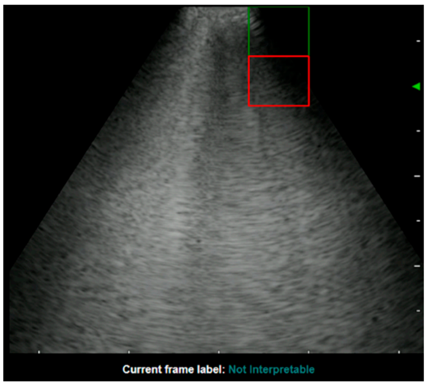

6. Examples of annotations

6.1. Example of *the label Lymph* node/blood *vessel*

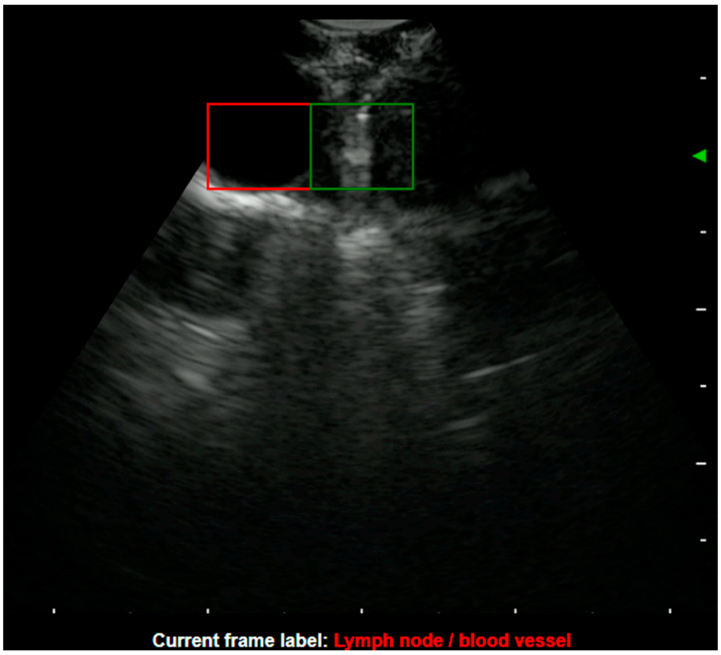

The Grad-CAM visualization shows the activation region (red and green squares) overlapping a clinically relevant structure. The annotation is classified as *Lymph node/blood vessel* according to the annotation criteria.

6.2. Example of *the label Lymph* node/blood *vessel*

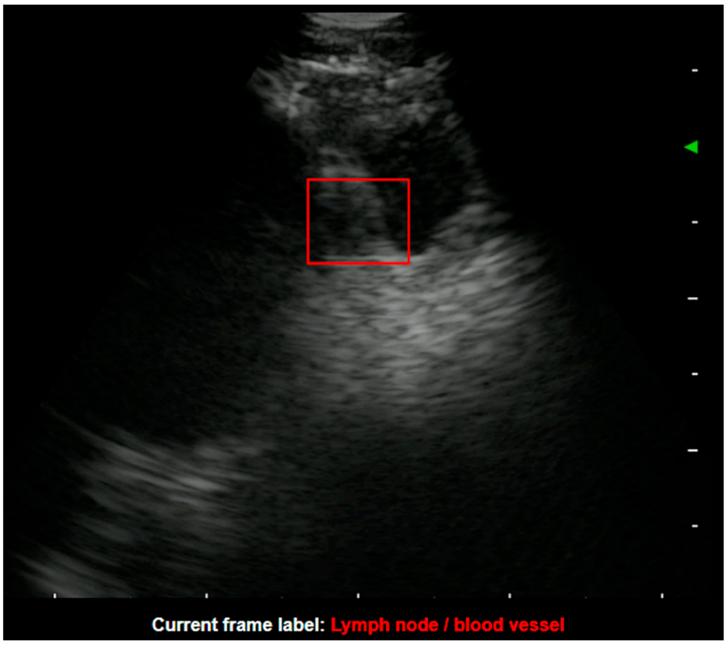

The activation region overlaps a portion of a clinically relevant structure, meeting the criterion that any degree of overlap with a lymph node or blood vessel warrants annotation as *Lymph node/blood vessel*, even when the overlap is minimal.

6.3. Example of the label artifact

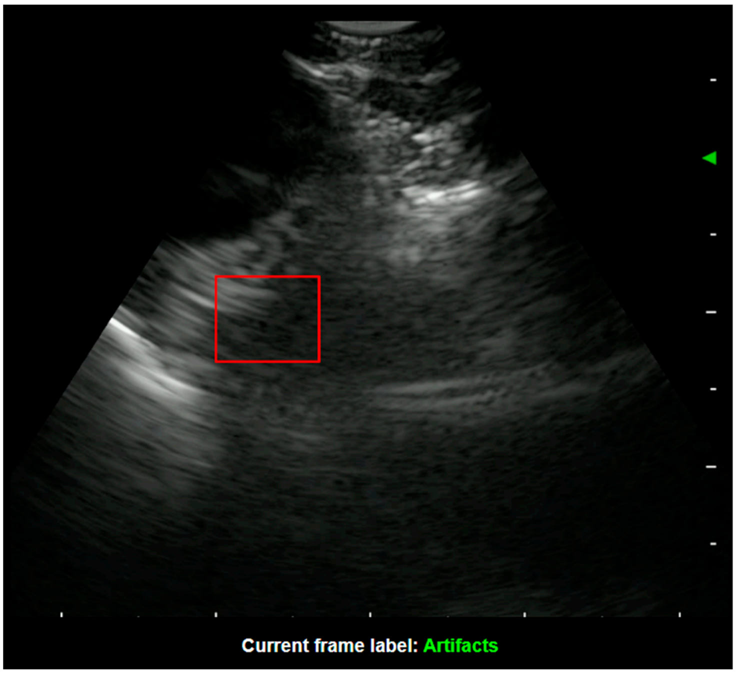

The activation region overlaps an ultrasound artifact. Because any overlap with an artifact takes precedence over other structures, and no part of the activation region aligns with a lymph node or vascular structure, the image is labelled as *artifact*.

6.4. Example of the label artifact

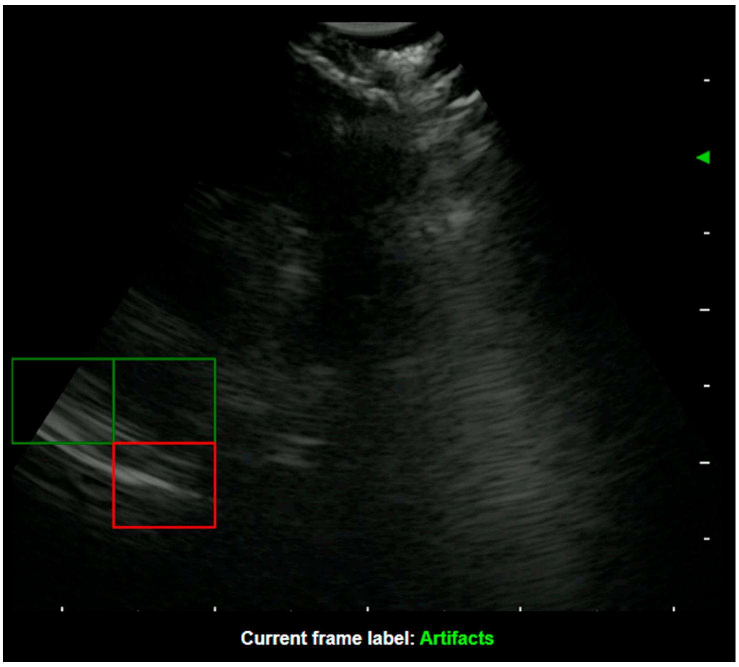

The activation region overlaps an ultrasound artifact. Because any overlap with an artifact takes precedence over other structures, and no part of the activation region touches a lymph node or blood vessel, the image is correctly labelled as *artifact*.

6.5. Example of the label other structure

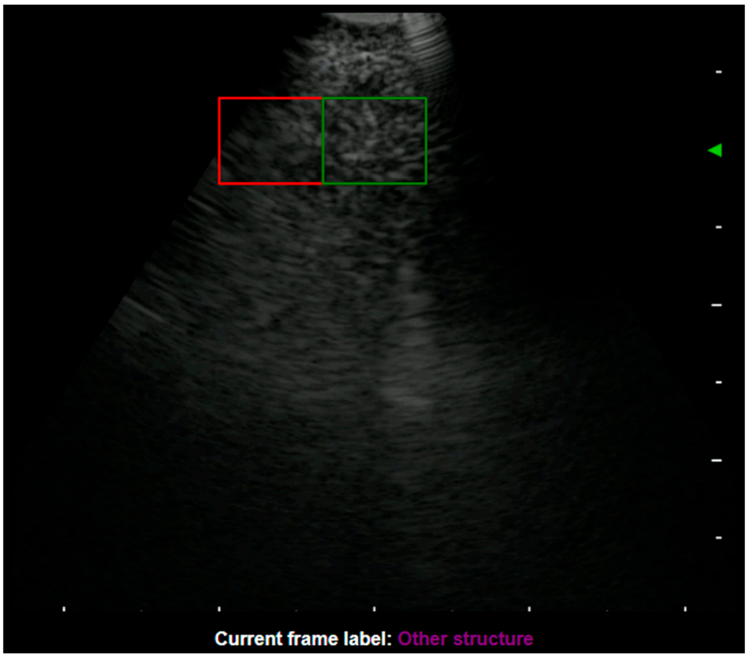

The activation region does not overlap a lymph node or blood vessel, and no part of the region aligns with an artifact. Instead, the activation region lies over other structures. Consequently, the image should be labelled as *other structure*.

6.6. Example of *the label* not *interpretable*

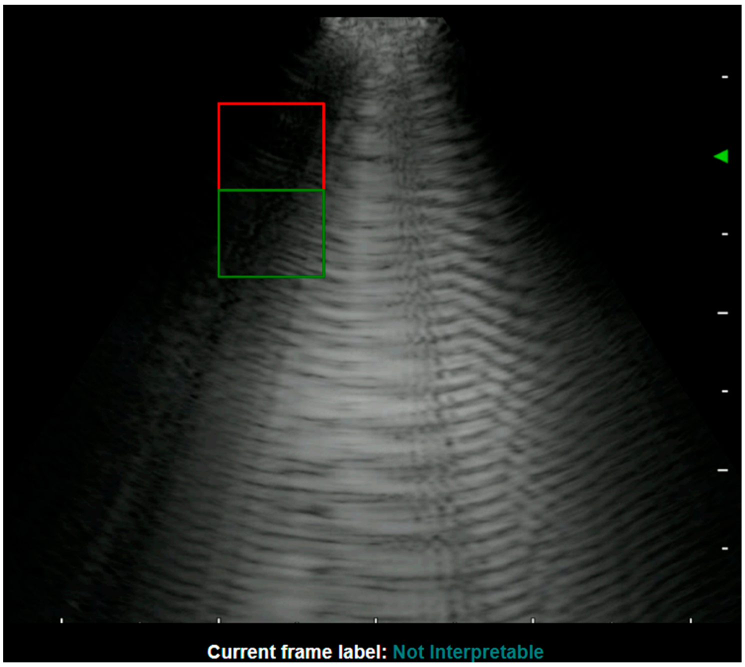

Most of the ultrasound sector exhibits severe global degradation, with no identifiable anatomical structures or orientation cues. The image is non-diagnostic, consequently, the image should be labelled as *not interpretable.*

7. Quality control

To ensure reliable and reproducible annotations, we recommend implementing the following steps:

7.1. **An initial joint annotation session** to ensure that all annotators share a consistent understanding of the guidelines and apply the categories uniformly.

7.2. **Independent annotation, where each** annotators independently labels assigned subsets of the dataset on individual image-level, following the annotation strategy defined in Section 4.

7.3. **Quantitative assessment of inter-annotator agreement**s using standard metrics (e.g., percent agreement, Cohen's or Fleiss' kappa) to assess reliability and identify systematic disagreements. Labels should be automatically accepted when there is complete or majority agreement (≥2 of 3 annotators, or >50% when more than three annotators are involved).

7.4. Images with complete disagreement (no majority label) should be reviewed by all participating annotators in a dedicated **consensus meeting.** The discussion should be guided by the predefined annotation rules. One final label should be assigned to their best effort, accepting dissent if the majority of annotators (>50%) come to an agreement during the discussion.
